# Reply: KRAS mutation in colorectal cancer metastases after adjuvant folfox for the primary

**DOI:** 10.1038/bjc.2012.420

**Published:** 2012-09-27

**Authors:** T Yoshino, Y Kawamoto, H Bando, K Tsuchihara

**Affiliations:** 1 Division of Translational Research, Research Center for Innovative Oncology, National Cancer Center Hospital East, Kashiwa 277-8577, Japan; 2 Department of Gastroenterology and Gastrointestinal Oncology, National Cancer Center Hospital East, Kashiwa 277-8577, Japan; 3 Department of Gastroenterology, Hokkaido University Graduate School of Medicine, Sapporo 060-8638, Japan

**Sir,**

We appreciate the comment from Dr Vauthey and colleagues (Vauthey *et al*, 2012) on our recently published article by Kawamoto *et al* (2012) titled ‘KRAS mutations in primary tumours and post-FOLFOX metastatic lesions in cases of colorectal cancer’. The *KRAS* mutation has been regarded as one of the major ‘driver mutations’ of colorectal cancer, and its frequency is high (approximately 40%) regardless of the Dukes stage (Andreyev *et al*, 1998). However, the prognostic and predictive usefulness of *KRAS* mutations is still controversial (Karapetis *et al*, 2008; Van Cutsem *et al*, 2009; Yoshino *et al*, 2012). Although the suggestion raised by Dr Vauthey that adjuvant FOLFOX may provide a selection pressure favouring a chemotherapy-resistant subset enriched for *KRAS* mutation is interesting, we should consider it carefully. We closely examined the seven cases of stage III colorectal cancer (metastases appeared ‘after’ FOLFOX therapy) in our data set and found that only two cases (28.6%) harboured *KRAS* mutations. Meanwhile, among the 14 cases of stage IV cancer (metastases appeared ‘before’ FOLFOX therapy), 10 cases (71.4%) harboured *KRAS* codon 12 mutations and 2 additional cases had *KRAS* A146V or *NRAS* Q61H mutations in both primary and metastatic lesions. Therefore, we could not conclude that *KRAS* mutations were enriched in the cases that relapsed ‘after’ adjuvant FOLFOX therapy in our data set.

However, we would like to emphasise that the frequency of *KRAS* mutations among the cases with ‘resectable’ metastases after FOLFOX therapy reported in our article was quite high. As we reported previously, the *KRAS* mutation frequency of oxaliplatin-refractory ‘unresectable’ metastatic colorectal cancer in our institute was 70 out of 159 (44.0%), which is equivalent to the rates described in the previous reports (Bando *et al*, 2011). We cannot deny that the mutant KRAS affected the biological features of metastasised tumours, and we plan further investigations.
